# Emergence time of CO_2_-forced European summer climate trends

**DOI:** 10.1038/s41598-026-44761-5

**Published:** 2026-03-23

**Authors:** Médéric St-Pierre, Joakim Kjellsson, Wonsun Park, Leonard F. Borchert, Mojib Latif

**Affiliations:** 1 GEOMAR Helmholtz Centre for Ocean Research Kiel, Kiel, Germany; 2 Faculty of Mathematics and Natural Sciences, Christian-Albrechts-University of Kiel, Kiel, Germany; 3 Rossby Centre, Swedish Meteorological and Hydrological Institute, SMHI, Norrköping, Sweden; 4 Center for Climate Physics, Institute for Basic Science, Busan, South Korea; 5 Department of Integrated Climate System Science, Pusan National University, Busan, South Korea; 6 Research Unit Sustainability and Climate Risk, Universität Hamburg, Hamburg, Germany

**Keywords:** Time of emergence, Hydrological cycle, Soil moisture, Near-surface temperature, Extreme summer, Climate sciences, Environmental sciences

## Abstract

This study investigates the time of emergence (ToE) of European summer climate trends in a warming world. We use a large ensemble of simulations performed with the Kiel Climate Model, in which atmospheric CO_2_ levels increase by 1% per year starting from pre-industrial concentration. The ToE for near-surface temperature, soil moisture, and the hydrological cycle highlights a relatively fast (20–40 years) emergence of near-surface temperature trends, while precipitation trends remain within the range of natural variability until the end of the 140-year-long simulation when CO_2_ levels quadruple. Soil moisture trends emerge after approximately 30 years in parts of the Mediterranean region, whereas in Western and Central Europe, they only emerge in the west after about 70 years when CO_2_ concentrations have doubled. Although many CO_2_-forced climate trends are not emerging over Europe, a comparison of 5,000 pre-industrial and 3,000 4xCO_2_ summers reveals statistically significant differences in the distribution of these summer variables: we find that the 1% driest summers are projected to be more extreme in a warmer 4xCO_2_ climate compared to the pre-industrial climate. This can have major implications for agriculture, as water shortages may become more severe during these extreme summers.

**Supplementary Information:**

The online version contains supplementary material available at 10.1038/s41598-026-44761-5.

## Introduction

Anthropogenic activities have warmed the globe since the pre-industrial period. This increase in global temperature caused many changes in the climate system, such as the behaviour of the hydrological cycle^1–3^ or the frequency of temperature-related extreme events^4^ in some regions. As a result, it has become increasingly important to understand the timing and extent of climate change impacts in Europe in order to implement effective and region-adapted climate change policies. A key metric for decision-makers is the Time of Emergence (ToE) of climate change^5–9^. The ToE is defined as the time required for a signal of climate change to emerge from the noise of natural variability^6,8^ around a base climate. In other words, the ToE is useful to identify regions that are or will be most affected by climate change and inform policymakers on where to implement adaptation strategies. In many cases, the ToE can be delayed if policymakers apply appropriate mitigation strategies. For instance, following a lower GHG emission scenario can greatly delay the ToE of many variables^5,10,11^.

Irrespective of whether the ToE is studied in observational or model datasets, analysis has mostly focused on near-surface temperature^8,12–16^, precipitation^6,10,17,18^, or both^7,9,19–21^. A general consensus across these studies is that climate change in surface temperature-related quantities emerges earlier compared to that of precipitation, climate extremes emerge as more extreme in a future warmer climate in many areas of the world, and mean surface temperature change emerges faster in tropical regions compared to extratropical regions. The latter is concerning, as tropical regions are usually less equipped to adapt to climate change^11^. Individual ToE studies have also covered a wide range of variables, such as sea level^22–24^, sea surface temperature^25^, soil moisture^26^, biochemical quantities^27–29^, and more. Here, we present a concise and unique study of the ToE for all hydrological quantities, soil moisture and near-surface temperature across Europe.

Climate change arises differently depending on the region, and one of the most affected continents is Europe, having warmed approximately twice as fast as the global average since the 1980s^30^, making it a key study region. We present results for all three European regions, defined by^31^; but given that the Mediterranean has been widely studied and is consistently projected to dry under a warmer climate^32–36^, our study focuses on western and central Europe, which has received less attention. The region is interesting because of its clear seasonal response to CO_2_ increase, with precipitation projected to increase in winter and decrease in summer, but with year-round soil moisture decline^37^.

The primary objective of this study is to demonstrate when and how the European summer climate will emerge from internal variability, with a particular emphasis on western and central Europe. Summers in this region are of particular interest because they are projected to experience reduced precipitation under a warmer climate, resulting in a large soil moisture decline during autumn^37^. Precipitation deficits can contribute to drought^38,39^, and low soil moisture may further intensify heat extremes^40^, which are more common in a warmer climate^4^. Our analysis quantifies the ToE and climate shift in summer soil moisture, hydrological cycle, and near-surface temperature over Europe, offering a detailed analysis of the statistical differences between pre-industrial and future climates. We also analyse the differences between the 1% driest summers in both climates to highlight the change of extreme summers. To perform this analysis, we use a Single Model Initial Conditions Large Ensemble (SMILE) (e.g.,^41,42^) of 100 simulations forced by a yearly 1% CO_2_ increase until initial pre-industrial concentrations quadruple. Our study shows that European summers will be significantly different in a future warmer climate, where CO_2_ is quadrupled, compared to a pre-industrial climate, whether the forced climate change signal emerges from internal variability or not.

## Data and method

### Model description

We use output from the Kiel Climate Model (KCM). The KCM^43^ uses the Hamburg atmospheric general circulation model version 5 (ECHAM5;^44^), developed at the Max Planck Institute (MPI). Most of the dynamics in ECHAM5 are solved in spectral space, while specific humidity and physics are solved in grid-point space. Our configuration uses a triangular truncation of T63 and a 1.88° horizontal grid (~ 200 km resolution). The vertical grid has 47 hybrid-sigma levels up to 0.01 hPa. The ocean-sea ice component is the Nucleus for European Modelling of the Ocean (NEMO;^45^) ocean-sea ice general circulation model with the Louvain-la-Neuve Ice Model version 2 (LIM2) sea ice model. Both models run on a tri-polar ORCA05 grid at a nominal resolution of 0.5° and 46 vertical depth levels. Coupling is handled by the Ocean Atmosphere Sea Ice Soil version 3 (OASIS3;^46^) coupler. Our study uses a version of the KCM with higher atmosphere and ocean model resolutions than the version described in^43^, and the NEMO version is 3.4. The KCM uses a bucket-type model with a single layer in the soil, as opposed to a more up-to-date multi-layered land model, which is a major limitation of this study. It also has a fixed leaf area index, and there is no interactive vegetation.

### Experiments

**Fig. 1:**
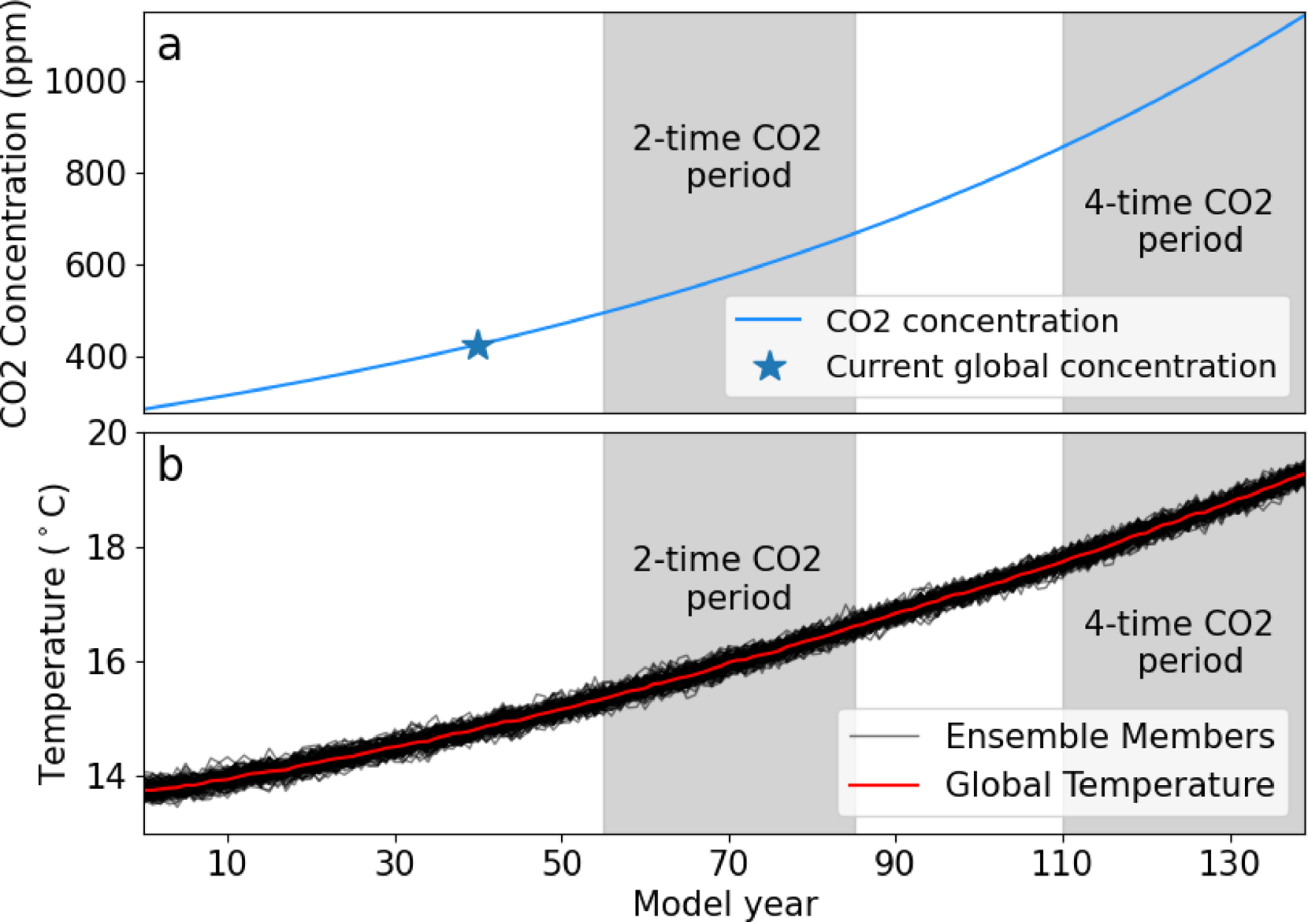
(**a**) Atmospheric CO_2_ concentration in the 1%-CO_2_ experiment with the Kiel Climate Model, and (**b**) global ensemble-mean temperature response (ºC) to the CO_2_ forcing. The pre-industrial CO_2_ concentration is set at 286.2 ppm. The black lines represent the 100 ensemble members. Years 55 to 85 are the 2×CO_2_ period, and the last 30 years are the 4×CO_2_ period.

We apply an atmospheric CO_2_ increase of 1% per year from pre-industrial levels to 100 simulations, thus creating the KCM large ensemble. Figure 1a, b shows the increase in CO_2_ over time and the response of the globally averaged near-surface (2 m) temperature in each ensemble member. The pre-industrial CO_2_ concentration is set at 286.2 ppm, is doubled (572.4 ppm) after 70 years, and quadrupled (1144.8 ppm) after 140 years. As a comparison, in the SSP585 scenario, which includes an increase in other greenhouse gases, the CO_2_ concentration by 2100 is ~ 1135 ppm^47,48^. The simulations differ only by their atmospheric and oceanic initial conditions, which we take every 50 years from a 5000-year pre-industrial control (piCTRL) run.

### Data treatment

**Fig. 2:**
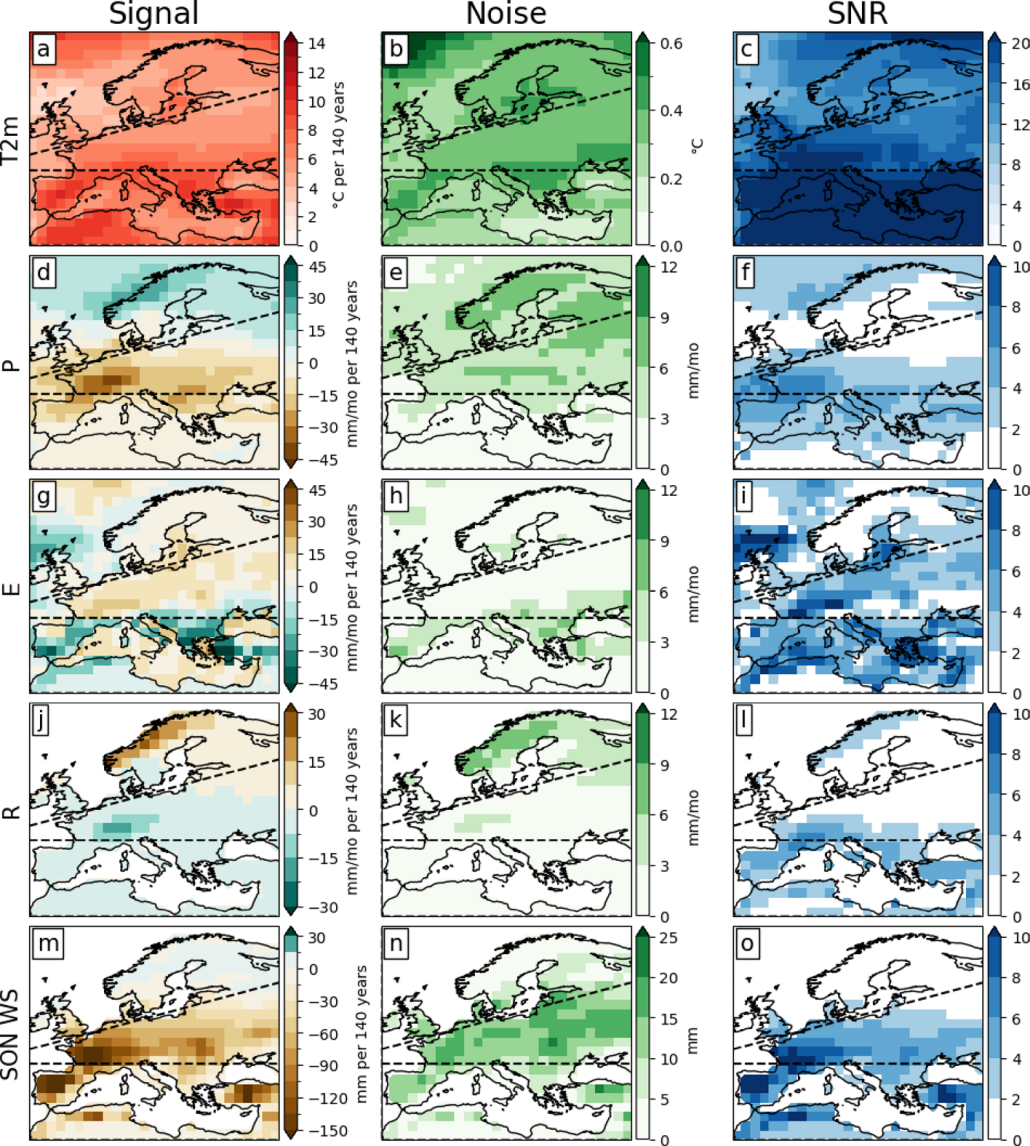
Ensemble-mean trend (left panels), standard deviation (middle panels), and signal-to-noise ratio (right panel). (**a**–**c**) JJA 2-meter temperature, (**d**–**f**) precipitation, (**g**–**i**) evaporation, (**j**–**l**) runoff, and (**m**–**o**) SON soil moisture. The dashed black line delimits the border of the European IPCC climate reference regions. The signal-to-noise ratio has no unit, and values larger than 2 are considered robust. Trends are for 140 years.

This paper focuses on the three European regions defined in the latest update of the IPCC climate reference regions^31^. These regions are Northern Europe (NEU), Western and Central Europe (WCE), and the Mediterranean (MED) and can be identified as the areas between the dashed lines in Fig. 2. Results are shown for the Northern Hemisphere summer months, which include June, July, and August (JJA). We found that the soil moisture responds to hydrological changes with an approximate lag of two to three months^37^, therefore the soil moisture results are displayed for the months of September, October, and November (SON). For simplicity purposes, when using the word “summer”, we also include SON soil moisture. The 30 years centred around year 70 of the 1%-CO_2_ runs is defined as the 2xCO_2_ period, and the last 30 years as the 4xCO_2_ period.

We perform a statistical analysis in Sect. 3.3 to study the differences between piCTRL and 4xCO_2_ summers. We use the two-sample Kolmogorov-Smirnov test (KS test) to determine if the piCTRL and 4xCO_2_ summer distributions are significantly different. The distributions are considered significantly different if the p-value of the KS-test is lower than 0.05 (95% confidence). A p-value of one would indicate that the distributions are identical. We remove the ensemble mean from the distributions when performing the KS test, which allows us to filter out the effect of the mean state from the significance test and assess the differences in the shape of the distributions instead. For instance, both distributions could be significantly different because there is a change in the number or type of extreme summers or because the range of possible summer climates increases (larger standard deviation) or decreases (lower standard deviation). This analysis is possible due to the large amount of data provided by the KCM SMILE.

### Time of emergence

This study calculates the ToE according to the “signal-to-noise” method^8,27^, which is the time at which the signal-to-noise ratio permanently exceeds a specific threshold. Various definitions of ToE have been used and are well detailed by^9^. The signal-to-noise method is defined as the time at which the CO_2_-forced climate emerges from the internal variability (σ) of a baseline climate. In this paper, we use a threshold of two standard deviations (2σ) of the summer annual mean. This is a strict criterion ensuring that the signal of climate change is felt every single year. Two standard deviations also represent the 95th percentile of the interannual variability for normal distributions. However, not all variables in this study are normally distributed. This study calculates the ToE for 2-meter temperature *(T2m)*, precipitation *(P)*, evaporation *(E)*, runoff *(R)*, and soil moisture *(WS)*. We determine the signal by fitting each variable *(Y)* at each grid cell to a linear regression for each ensemble member of the 1%-CO_2_ experiment:

1 $${Y\left(t\right)}_{m}={\alpha}_{m}t+{\beta}_{m}$$

where$m$ is the individual ensemble member, $t$ is the year since the start of the simulation, ${\alpha}_{m}$ is the linear trend, and ${\beta}_{m}$ is the intercept. The trends are not necessarily linear, thus this assumption is not perfect. The baseline climate is the 5,000-year piCTRL run. The σ is defined as the standard deviation of the piCTRL yearly JJA variables, leading to the following ToE definition:

**Fig. 3:**
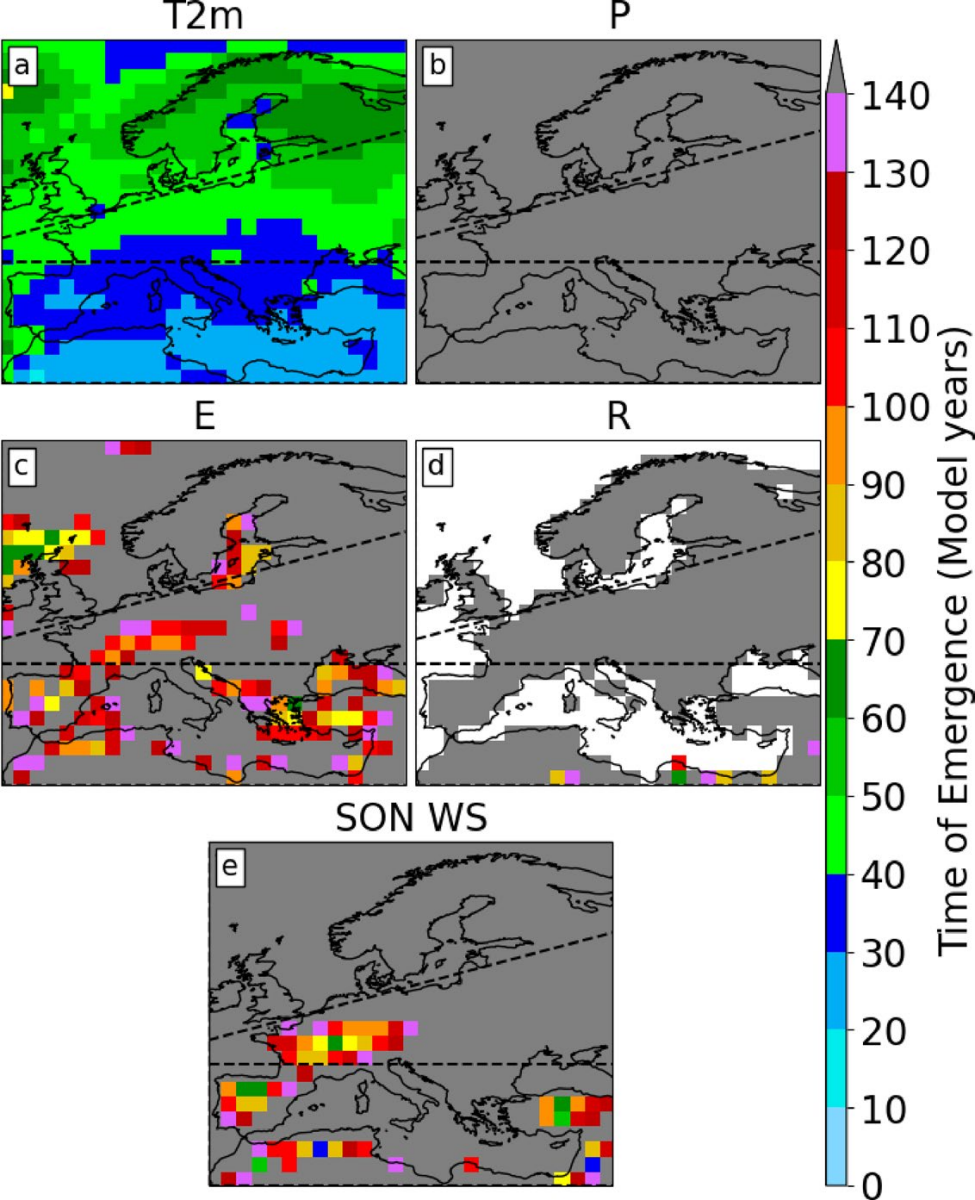
Ensemble-median Time of Emergence for (**a**) JJA 2-meter temperature, (**b**) precipitation, (**c**) evaporation, (**d**) runoff, and (**e**) SON soil moisture. The dashed black line delimits the border of the European IPCC climate reference regions.

2 $${ToE}_{m}=\frac{2\times\sigma}{{\alpha}_{m}}$$

This equation holds for linear trends. The ensemble median *ToE* is obtained by calculating the median of the individual ensemble members.

## Results

### CO_2_-forced climate trends, noise, signal-to-noise ratio

Analysing the ensemble-mean trend (signal), ensemble standard deviation (noise), and signal-to-noise ratio (SNR; signal/noise) (Fig. 2), we find robust warming trends in T2m, ranging from 3 to 12 °C for 140 years across all European regions (Fig. 2a). The T2m trends are the most robust among all analysed variables, with SNR exceeding 12 in most of Europe and 20 for the whole MED (Fig. 2c). We find significant negative precipitation trends (SNR > 2, Fig. 2d, f) over the majority of WCE and northern MED. The largest negative precipitation trends (< -45 mm/mo) and the largest SNR (> 6) are in the western part of WCE. In Northern Scandinavia, JJA precipitation trends are positive (> 22 mm/mo), and the SNR there exceeds 2. We find robust negative soil moisture trends (< -150 mm) in both MED and southern WCE (Fig. 2m), with SNR greater than 4 in most of both regions and values surpassing 10 in areas such as Spain, Portugal, France, and Turkey (Fig. 2o). Evaporation trends (Fig. 2g, i) are positive (negative) and robust over WCE (MED). Positive runoff trends (Fig. 2j, l) are robust in northern Norway and Sweden, whereas negative trends are robust over the Alps.

**Fig. 4:**
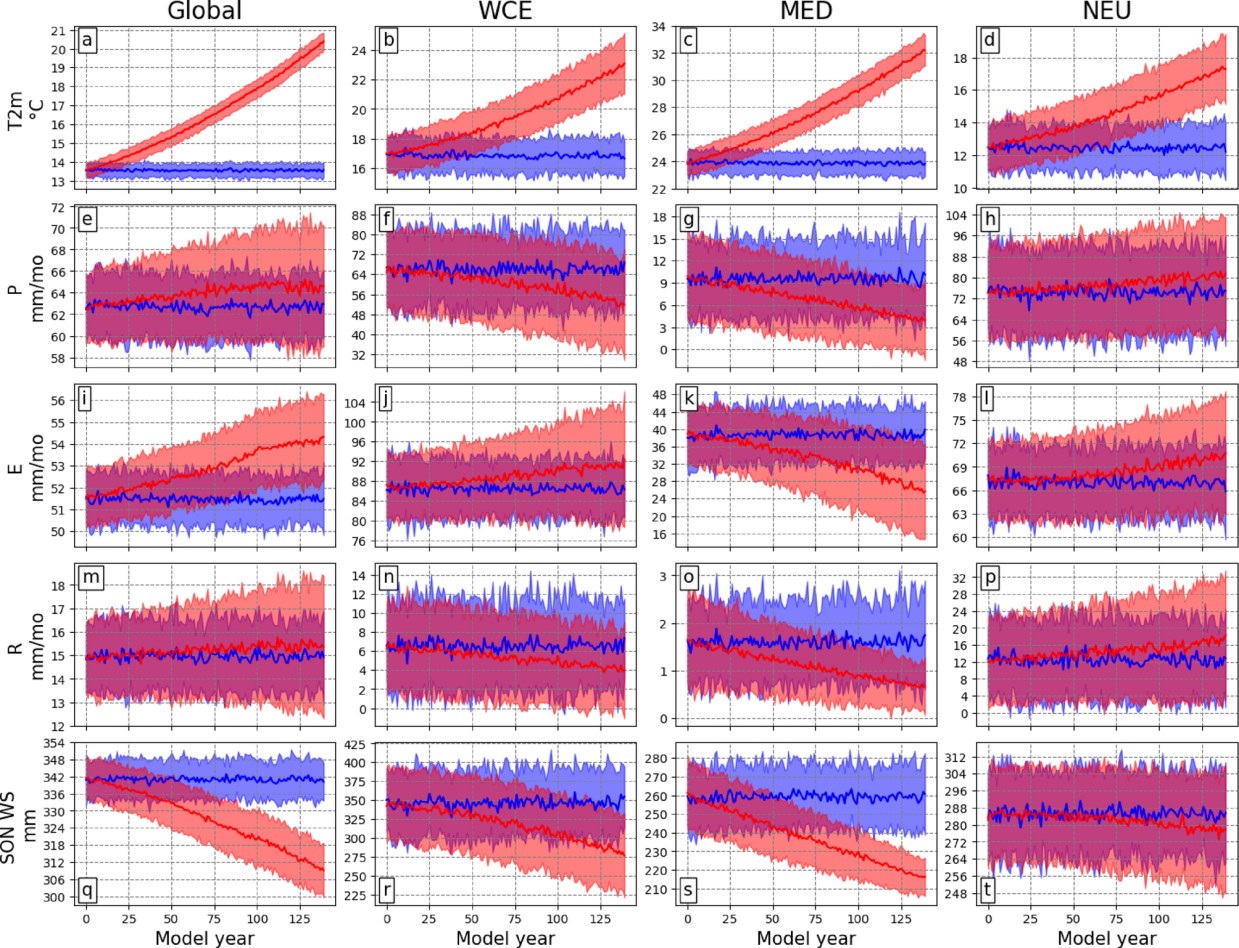
Ensemble-median time series and uncertainty ranges. (**a**–**d**) JJA 2-meter temperature, (**e**–**h**) precipitation, (**i**–**l**) evaporation, (**m**–**p**) runoff, and (**q**–**t**) SON soil moisture. The time series are calculated **only for land grid cells** at the global scale and for the three European regions. The **blue** and **red** lines represent the ensemble medians for the piCTRL and 1%-CO_2_ simulations, respectively. Shading shows the model spread (2σ). The 5000-year piCTRL run was divided into 35 periods of 140 years. The time at which the 1%-CO_2_ trend crosses the piCTRL blue shading indicates the ToE of the CO_2_-forced trend.

### Time of emergence

In the ensemble median, the ToE for T2m across Europe is less than 70 years (Fig. 3a). In the MED region, the ToEs range from 20 to 40 years and are the shortest in Europe, compared to NEU, where changes are emerging after 50 to 70 years. While T2m changes emerge relatively quickly, other variables exhibit ToEs longer than the 140-year simulations. Precipitation (Fig. 3b) and runoff (Fig. 3d) changes, for instance, are, on average, not emerging across Europe by the time of CO_2_ quadrupling. Evaporation trends (Fig. 3c) are emerging over a large area of MED and western WCE, but not before year 70, when CO_2_ concentration doubles. The same is true for the soil moisture trends in western WCE. Parts of Spain, Turkey, and northern Africa show ToEs of less than 70 years for soil moisture. Generally, the ToE of any variable is lower (Fig. 3) where the SNR is large (Fig. 2c, f, i, l, o) across the ensemble members. However, except for T2m, a high SNR (> 2) does not automatically relate to emerging trends. For instance, precipitation (Fig. 2d, f) and runoff (Fig. 2j, l) exhibit robust trends (SNR > 6) in some regions, although the signal does not emerge from the internal variability (Fig. 3b, d).

We illustrate area-median ToEs using time series and uncertainty envelopes (Fig. 4), when the ensemble median of the 1%-CO_2_ experiment exceeds twice the standard deviation (shading) of the piCTRL simulation. Global changes are emerging early compared to those in Europe. The ToE for global T2m is the earliest, occurring at approximately 15 years, followed by WS and E, at 50 and 80 years (Fig. 4a, i, q), respectively. P (Fig. 4e) and R (Fig. 4m) do not emerge. In Europe, CO_2_-forced T2m changes emerge relatively early across all regions (Fig. 4b-d), with the trend in NEU emerging last, after 70 years. Precipitation does not emerge from the internal variability in all regions (Fig. 4f-h), consistent with the spatial patterns (cf. Fig. 3b). In the WCE and MED regions, the precipitation trend almost exceeds the noise after reaching 4xCO_2_ (Figs. 4f, g). Evaporation only emerges in the MED region after 110 years (Fig. 4k), while the ToE for soil moisture in SON is 60 years in MED (Fig. 4s) and 125 years in WCE (Fig. 4r). The MED is the only region where the uncertainty range decreases in the 4xCO_2_ climate compared to the piCTRL period for precipitation (Fig. 4g), runoff (Fig. 4o), and soil moisture (Fig. 4s), indicating a strong confidence in the drying of the Mediterranean region.

**Fig. 5:**
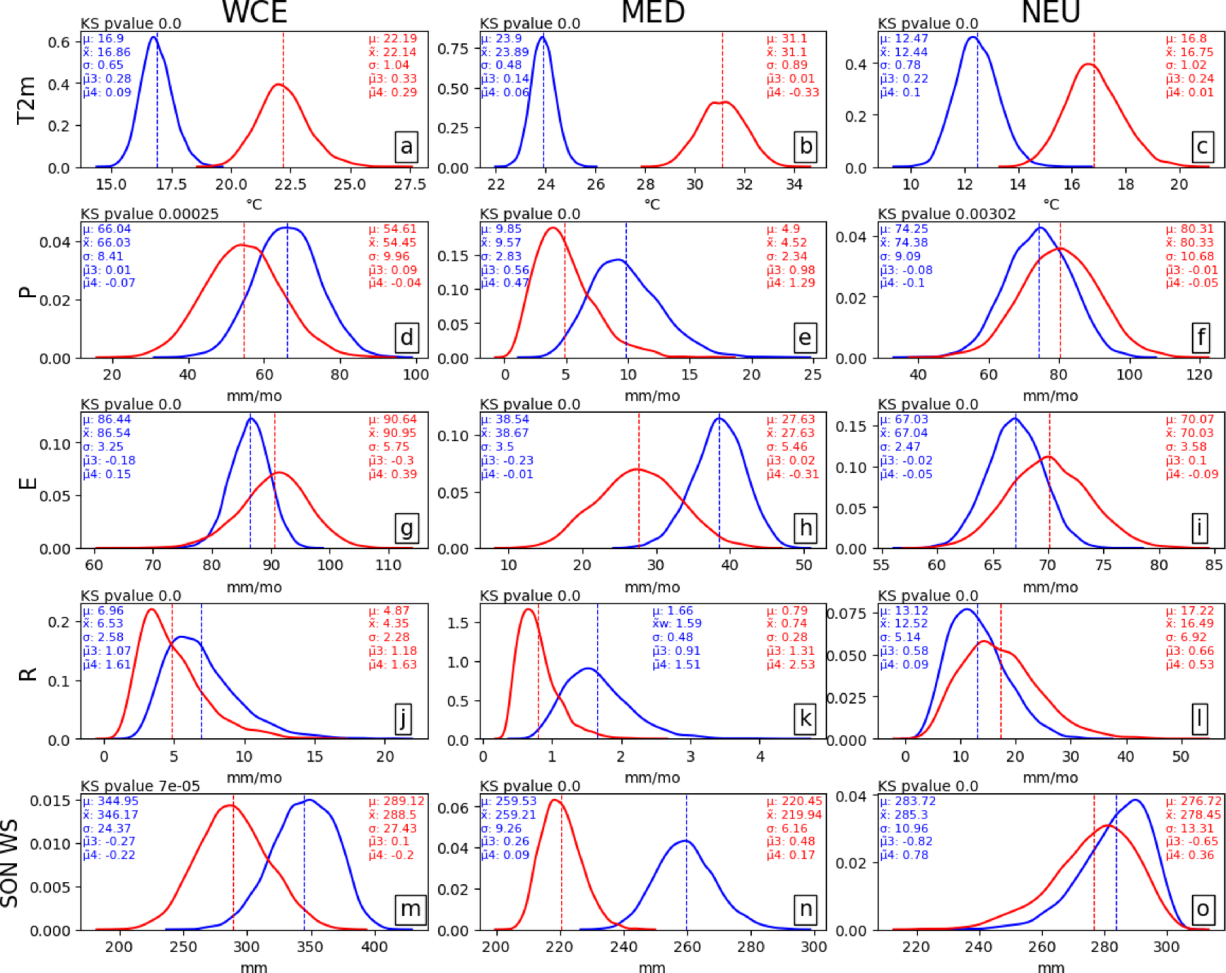
Distribution of 5000 piCTRL summers (blue) and the 3000 4xCO_2_ summers (red) for (**a**–**c**) JJA 2-meter temperature, (**d**–**f**) precipitation, (**g**–**i**) evaporation, (**j**–**l**) runoff, and (**m**–**o**) SON soil moisture. The dashed lines represent the mean of the distributions. Mean ($\boldsymbol{\mu}$), median ($\stackrel{\sim}{\boldsymbol{x}}$), standard deviation ($\boldsymbol{\sigma}$), skewness (${\stackrel{\sim}{\boldsymbol{\mu}}}_{3}$), and kurtosis (${\stackrel{\sim}{\boldsymbol{\mu}}}_{4})$ of the distribution are displayed in their respective colours.

### Changes in climate distributions

Our statistical comparison of 5,000 piCTRL and 3,000 4xCO_2_ summers (Fig. 5) shows that even if some variables do not emerge from the internal variability, all quantities are significantly different (p-value < 0.05) in a 4xCO_2_ climate compared to a piCTRL climate, indicating a significant emergence of all extreme summers under quadrupling of CO_2_.

We analyse the differences using the statistics of the distributions. For instance, the standard deviation (STD) of most variables increases across all three regions under increasing CO_2_ forcing (with the exception of P, R, and WS in MED and R in WCE), resulting in broader distributions. This increase in STD indicates a larger variability and a tendency towards more extreme JJA values in a 4xCO_2_ climate. Some distributions, such as P, R, and WS in MED (Fig. 5e, k, n), show increased positive skewness compared to the piCTRL climate, meaning larger high extremes. In the case of P (Fig. 5e) and R (Fig. 5k) and in contrast to the piCTRL run, the mean in the 4xCO_2_ climate in MED is very close to zero in summer, thereby limiting the occurrence of low extreme values. The runoff distributions (Fig. 5j-l) display positive skewness in both the piCTRL and 4xCO_2_ climates, suggesting larger high extreme runoff values compared to low values. In the piCTRL run, WS shows negative skewness in WCE (Fig. 5m) and NEU (Fig. 5o), indicating that there are more dry extremes compared to wet extremes. The most likely reason is that the soil is already wet in the piCTRL, limiting its ability to take more water. The additional water would run off, preventing the occurrence of extreme wet events. While WS in NEU retains its negative skewness in the 4xCO_2_ climate, the distribution in WCE moves towards a more normal distribution, leading to equally extreme dry and wet soil moisture. However, the overall WS drying of the WCE region results in more severe dry extremes and less severe wet extremes. The kurtosis increases in WCE for T2m (Fig. 5a) and E (Fig. 5g), in MED for P (Fig. 5e) and R (Fig. 5k), and in NEU for R (Fig. 5l), showing a rise in the frequency of extreme low and high values. In contrast, the kurtosis decreases in WCE for WS (Fig. 5m) and in MED for T2m (Fig. 5b) and E (Fig. 5h), suggesting an overall reduction in the occurrence of extreme values. Other distributions remain relatively unchanged.

We identify numerous changes in extremes from the comparison of the distributions. For instance, the T2m distributions (Fig. 5a, c) show that what is considered an extreme warm summer in WCE and NEU during the piCTRL period would be regarded as an extreme cold summer in the 4xCO_2_ climate. Moreover, in the MED region, 4xCO_2_ summer T2m would be completely unknown to the pre-industrial climate (Fig. 5b). In the case of P, E and R in MED (Fig. 5e, h and k) as well as WS in WCE (Fig. 5m), the mean values in the 4xCO_2_ period would correspond to extreme low values in the piCTRL climate. Furthermore, the mean 4xCO_2_ WS in MED (Fig. 5n) falls outside the internal variability of the piCTRL period, meaning that pre-industrial summers considered very dry in MED are extremely wet in the 4xCO_2_ climate.

**Fig. 6:**
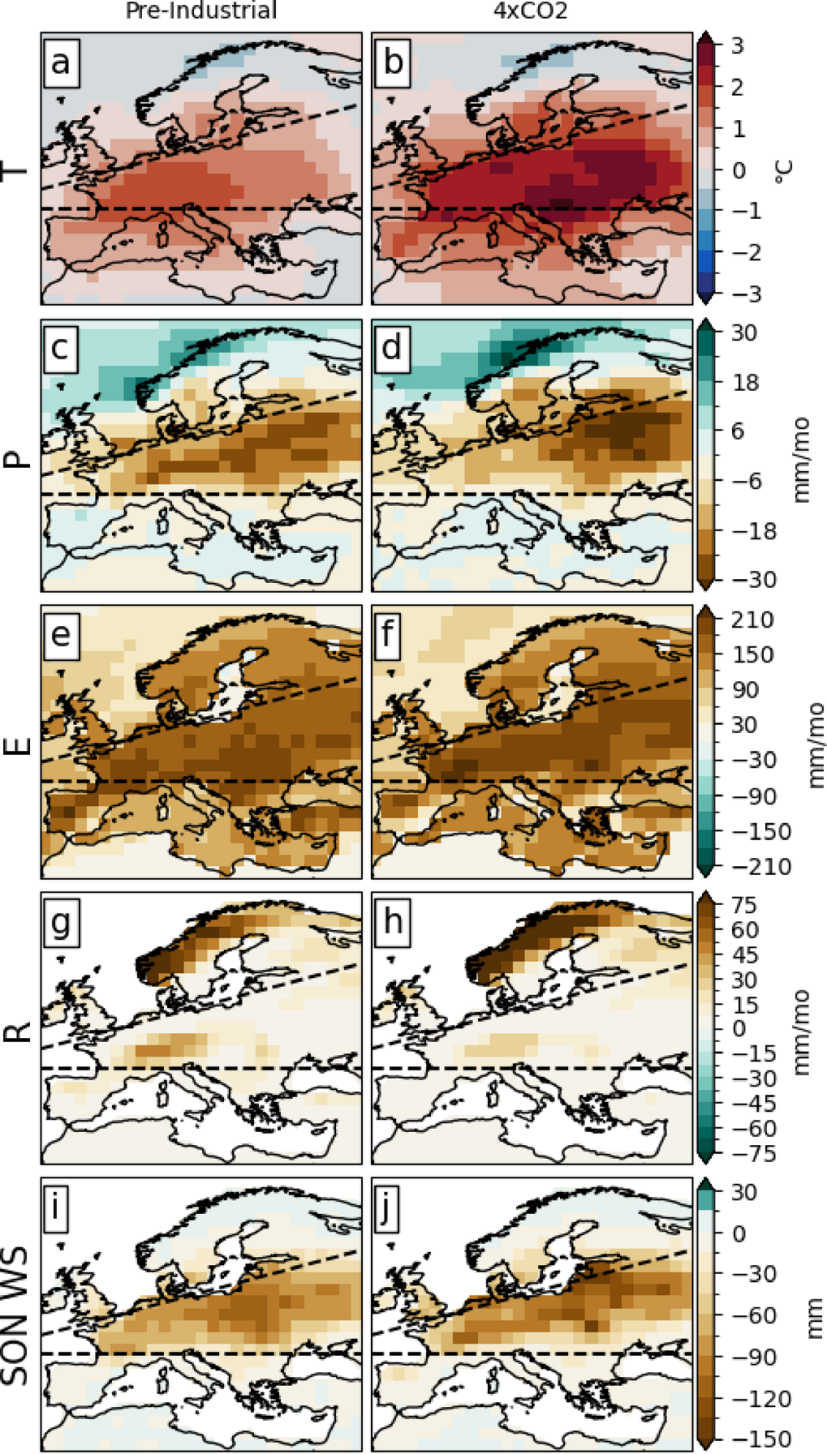
JJA (**a**,**b**) 2-meter temperature, (**c**,**d**) precipitation, (**e**,**f**) evaporation, (**g**,**h**) runoff, and (**i**–**j**) SON soil moisture anomalies associated with the 1% driest falls (SON) in WCE. The left panels display the results for the piCTRL run, and the right panel for the 4xCO_2_ period. The dashed black line delimits the border of the European IPCC climate reference regions.

### Changes in WCE extreme dry summers

Due to WS responding to hydrological change with a 2–3 month delay, we compare summers leading to extremely dry falls (see Sect. 2.3) in the piCTRL and the 4xCO_2_ climates (Fig. 6). The 1% driest SON values (or 1st percentile), identified based on the average SON soil moisture across all grid cells within WCE, were selected. This selection includes 50 SON values from the piCTRL simulation and 30 SON values from the 4xCO_2_ climate. T2m, P, E and R are displayed for JJA. Subsequently, the mean climate for each variable was subtracted from the mean of the 1st percentile to obtain anomalies.

The spatial WS patterns of an extremely dry summer in the piCTRL and 4xCO_2_ climates are similar (Fig. 6i, j). However, in the 4xCO_2_ period, the dry WS anomalies shift slightly northward and become generally more intense. The centre of the warm T2m anomalies shifts from western WCE in the piCTRL (Fig. 6a) to eastern WCE in the 4xCO_2_ climate (Fig. 6b), expanding to cover a larger portion of Europe and being generally more pronounced in the 4xCO_2_ period. The precipitation patterns also change with CO_2_ increase. In the piCTRL run, negative precipitation anomalies are evenly distributed across the WCE region, compared to the 4xCO_2_ climate, where they become more intense and are concentrated in the northeastern part of WCE (Fig. 6c, d). Notably, the maximum precipitation anomalies observed in western WCE in the piCTRL run are absent in the 4xCO_2_ period. Overall, extreme dry summers, relative to their mean climate, become more intense in the 4xCO_2_ climate, with the most significant contrast found in the northeastern part of the WCE region.

## Discussion and conclusion

This study uses the Kiel Climate Model (KCM) large ensemble consisting of 100 simulations, where atmospheric CO_2_ concentrations are increased by 1% per year until quadrupled after 140 years. We use the ensemble to determine the JJA trends and Time of Emergence (ToE) of the complete European hydrological cycle, soil moisture, and near-surface 2-m temperature.

We find robust trends (SNR > 2) across the ensemble members over Europe for all analysed variables. T2m exhibits the most robust trends, with SNR values exceeding 20 in many areas. The MED and WCE regions experience important negative trends in P and WS, with SNR values well over 6 in several areas. Despite the strong CO_2_ forcing and robust trends, the signal of climate change is not emerging for European precipitation according to the calculated ToEs. However, in contrast to the ensemble median ToE shown in Fig. 3b, a few ensemble members showed emergence from the internal variability for precipitation in western WCE (Supp. Fig. 1b), but only after more than a century. The WS signal emerges in western WCE after 70 years. WS also emerges in most of MED, with the shortest ToEs being on the order of 30–70 years in some areas. T2m ToEs are the smallest among all variables, ranging from 20 to 40 years in MED to 40–70 years in NEU. Temperature trends emerging first are in line with previous findings^21^.

Comparing global and regional JJA ToEs, it is evident that the climate change signal emerges earlier at the global scale, agreeing with previous research^7^. This earlier emergence is expected, as^49^ showed that internal variability is larger at the regional scale compared to the global scale. Among the analysed variables, T2m is the only variable emerging from internal variability within a few decades in all three European regions. WS emerges after approximately 60 years in MED and 120 years in WCE. Although the decreasing precipitation trends in WCE and MED are not emerging from internal variability, they are very close to emergence after CO_2_ concentrations have quadrupled. This indicates that summers in a warmer world might still be very different from a pre-industrial world, even if their mean characteristics remain in the range of internal variability. In this study, we use the signal-to-noise ToE calculation method^8,27^, and we choose a restrictive threshold of two standard deviations of the annual mean. This threshold ensures that the signal of climate change is detectable in a single year. Using a different threshold like two standard deviations of the decadal to three-decadal mean^6^ or simply one standard deviation would result in an earlier ToE. The signal-to-noise method is typically applied to near-Gaussian distributions. However, as shown in Fig. 5, some variables, such as soil moisture and runoff, are not normally distributed. A different ToE calculation method, such as the Kolmogorov-Smirnov test, as used in^9^, for the calculation of the ToE may display different results.

The “almost” emergence of many JJA quantities (Fig. 4) led us to compare the distributions after removing their respective climatology from the piCTRL run and 4xCO_2_ period. We find them all to be significantly different at the 95% confidence level, regardless of emergence. A notable finding is that distributions in the 4xCO_2_ climate generally broaden, indicating a tendency towards more extreme JJA values. In some cases, values that were previously considered positive (negative) extremes in the piCTRL climate are categorised as negative (positive) extremes in the 4xCO_2_ period. While 4xCO_2_ is an extremely strong forcing, most of these distributions are still significantly different in the 2xCO_2_ climate (Supp. Fig. 2), and when globally averaged T2m reaches a 2 °C warming (Supp. Fig. 3). This is true for the mean state and the shape of the distribution.

Lastly, our study shows that under a 4xCO_2_ climate, extreme JJA exhibits more intense anomalies across all variables but runoff. The centre of these anomalies shifts from western to eastern WCE for both T2m and P. Overall, extreme dry summers in WCE become more intense in a 4xCO_2_ climate, with the most significant changes occurring in the northeastern part of the region. The reduced WS in a 4xCO_2_ climate may contribute to the warmer anomalies through the soil moisture feedback^40,50^. The limited availability of soil moisture under dry and warm conditions restricts latent heat transfer, thus increasing the soil temperature. If the soil temperature exceeds that of the surface air, sensible heat is transferred from the soil to the atmosphere, increasing the T2m and further drying the soil.

It is important to note that this study investigated CO_2_-forced trends by applying a single model and scenario, the KCM and the CO_2_ concentration pathway (1%-CO_2_), therefore our results do not include model and scenario uncertainty. Mean-state biases are large in the KCM, as in most state-of-the-art climate models, which may also bias ToEs. Over the majority of Europe, we find a cold JJA T2m bias and a negative precipitation bias (Supp. Figs. 4, 5). Lastly, the land model in the KCM has its limitations, as it uses a bucket-type model with a single layer in the soil. Using a multi-layered land model, where more land processes are resolved, might give different results. For instance, a possible shorter ToE for the upper soil layers due to reduced moisture retention.

The 1%-CO_2_ experiment represents a strong external forcing. However, by year 140 of the experiment, the CO_2_ concentrations are comparable to those projected by 2100 under high-emission scenarios such as SSP8.5^48,51^. This experiment is an idealised design intended to isolate the effect of greenhouse gas increase on the climate system. It does not directly account for changes in other greenhouse gases, such as methane, nor does it include important processes like land-use changes. More realistic future scenarios based on projected global socioeconomic developments, such as the Shared Socioeconomic Pathway^52^, may give different results. Nonetheless, our study demonstrates that a yearly 1%-CO_2_ increase might not necessarily lead to emerging summer trends over Europe, but it will lead to a significantly different and potentially unknown summer climate and extremes in Europe compared to the pre-industrial period. These findings may support policymakers in developing strategies to reduce greenhouse gas emissions, which have been shown to delay the ToE of climate trends^5^.

## Supplementary Information

Below is the link to the electronic supplementary material.

Supplementary Material 1

## Data Availability

The dataset analysed during this study is large and will be made available by the first author if requested. Only the precipitation data and the code used for the calculations are available at the following repository: [https://doi.org/10.5281/zenodo.17662629].
