# Supplementary material for: Emergence time of CO2-forced European summer climate trends
Source: Sci Rep. 2026 Mar 23;16:9707. doi: 10.1038/s41598-026-44761-5 (PMC13013919; doi:10.1038/s41598-026-44761-5)
Supplement: Supplementary file 1 — Supplementary Material 1 [file 41598_2026_44761_MOESM1_ESM.pdf]

Emergence time of CO_2-forced European summer climate trends

Médéric St-Pierre[1], Joakim Kjellsson[1,2,3], Wonsun Park[4, 5], Leonard Borchert[6], Mojib Latif[1,2]

[1]GEOMAR Helmholtz Centre for Ocean Research Kiel, Kiel, Germany
[2]Christian-Albrechts-University of Kiel, Faculty of Mathematics and Natural Sciences, Kiel, Germany
[3]Swedish Meteorological and Hydrological Institute, Rossby Centre, SMHI, Sweden
[4]Center for Climate Physics, Institute for Basic Science, Busan, South Korea
[5]Department of Integrated Climate System Science, Pusan National University, Busan, South Korea
[6]Research Unit Sustainability and Climate Risk, Universität Hamburg, Hamburg, Germany

Correspondence *to*: Médéric St-Pierre (mstpierre@geomar.de)

Supplementary Information. This paper was submitted in Springer Nature Scientific Report

Supplement

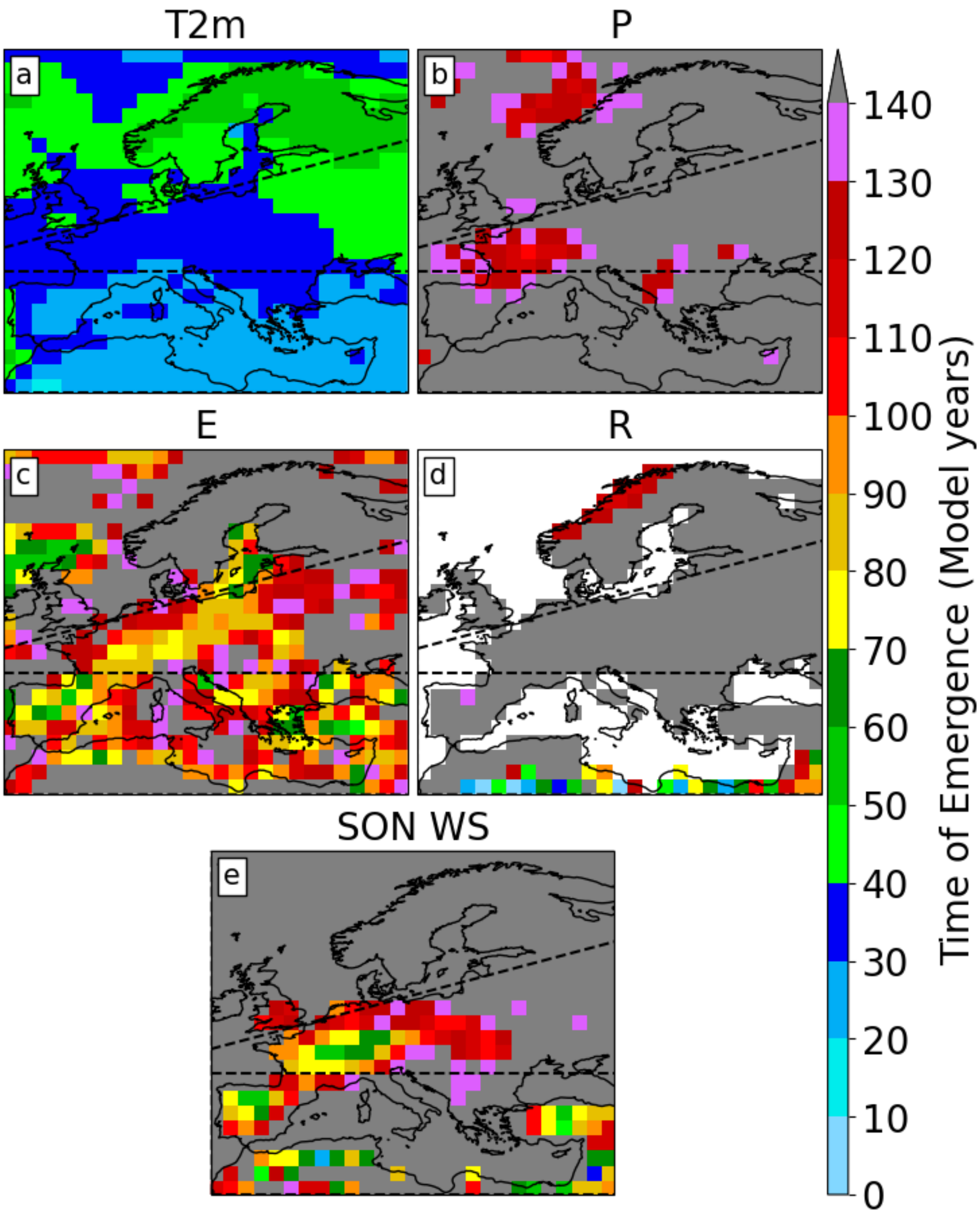

Supplementary Fig. 1 Ensemble-minimum Time of Emergence for (a) JJA 2-meter temperature, (b) precipitation, (c) evaporation, (d) runoff, and (e) SON soil moisture. The dashed black line delimits the border of the European IPCC climate reference regions.

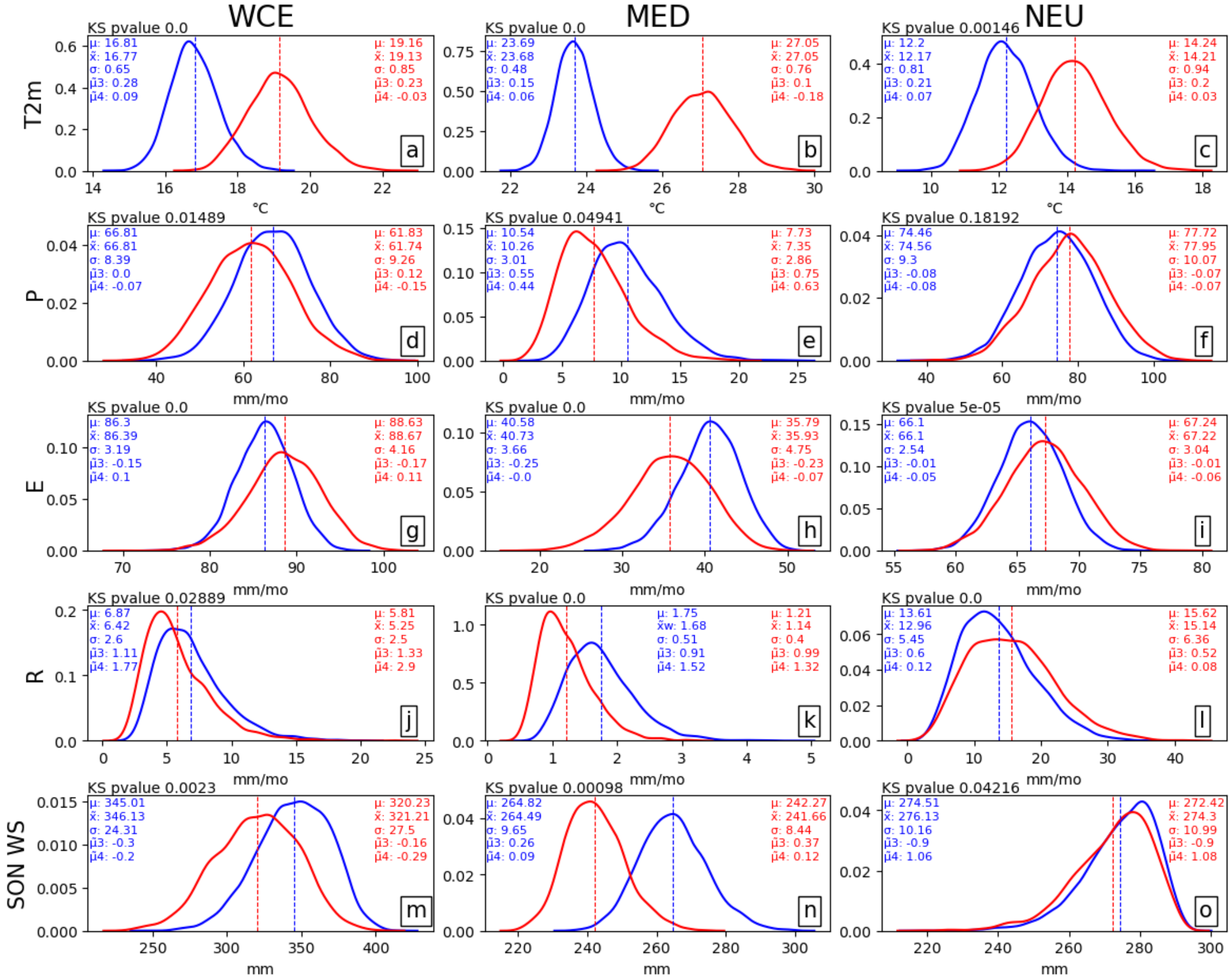

Supplementary Fig. 2 Same as Figure 5 but for 3000 $2xCO_2$ Summers

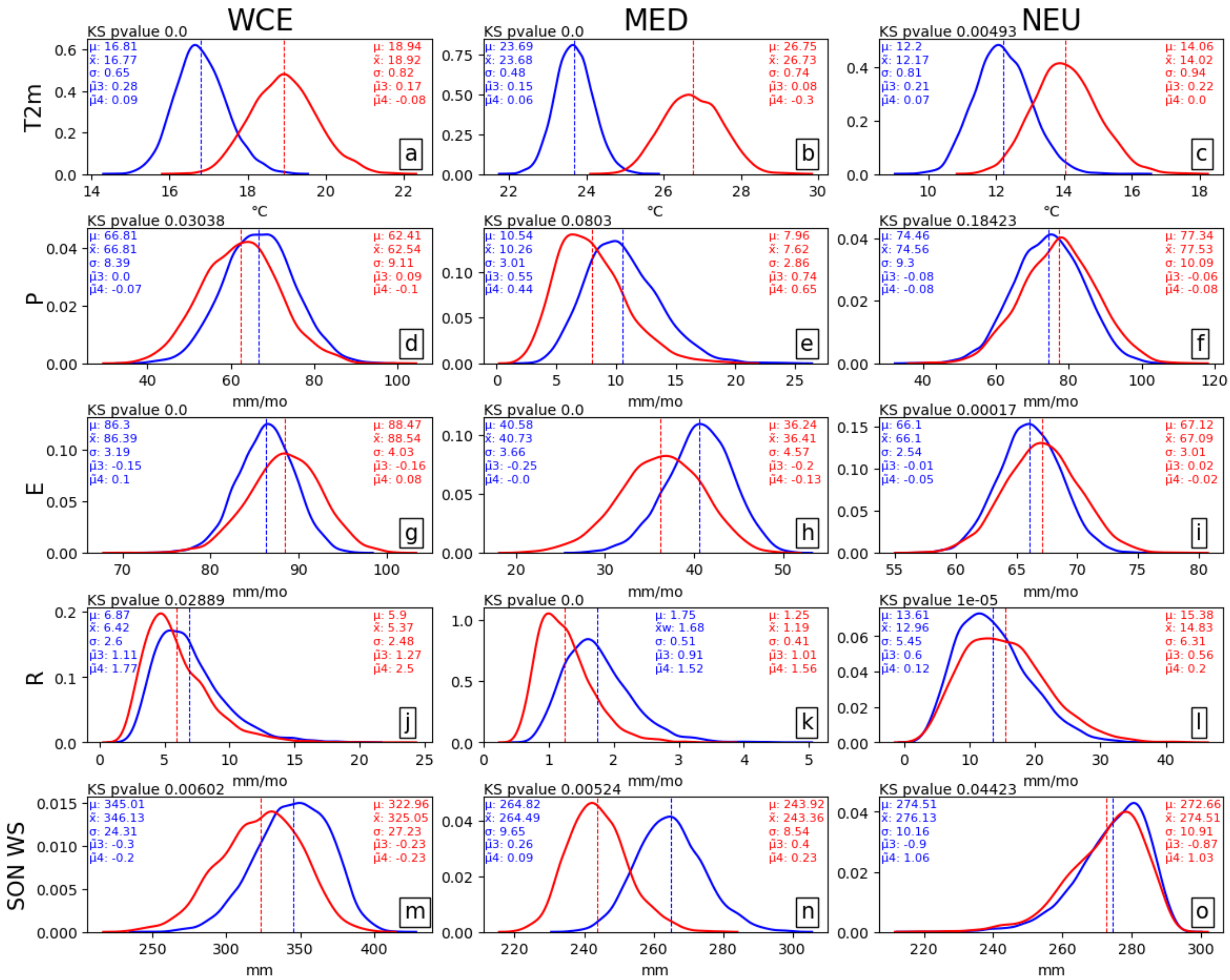

Supplementary Fig. 3 Same as Figure 5 but for 3000 Summers after 2°C warming.

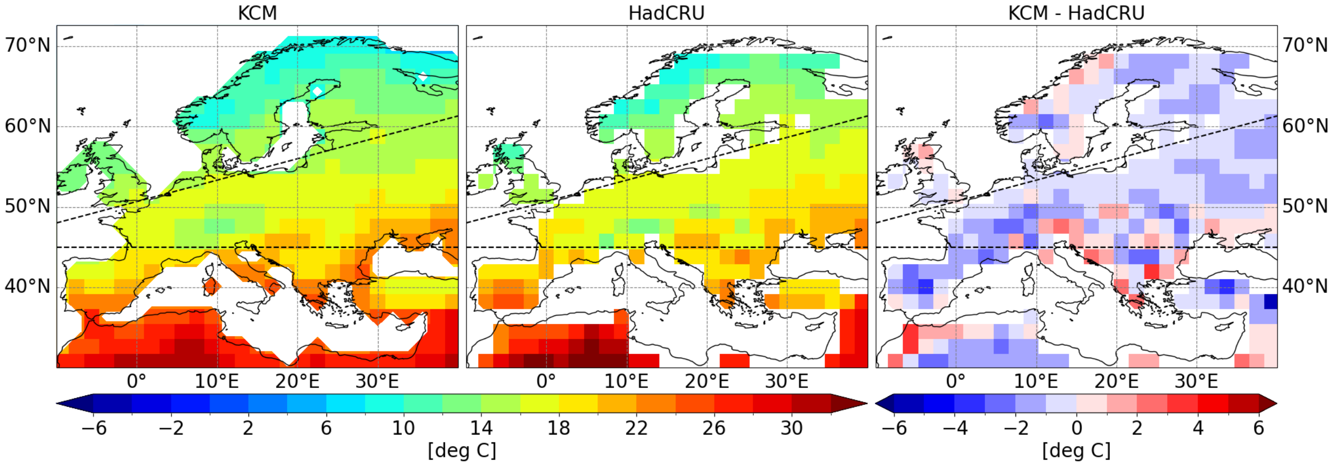

Supplementary Fig. 4 JJA average 2-meter temperature from (a) the reference period in the KCM (year 1-30), (b) the HadCRUT4 data set (year 1981-2010) and (c) bias (KCM – HadCRUT4). Units are in degree Celsius

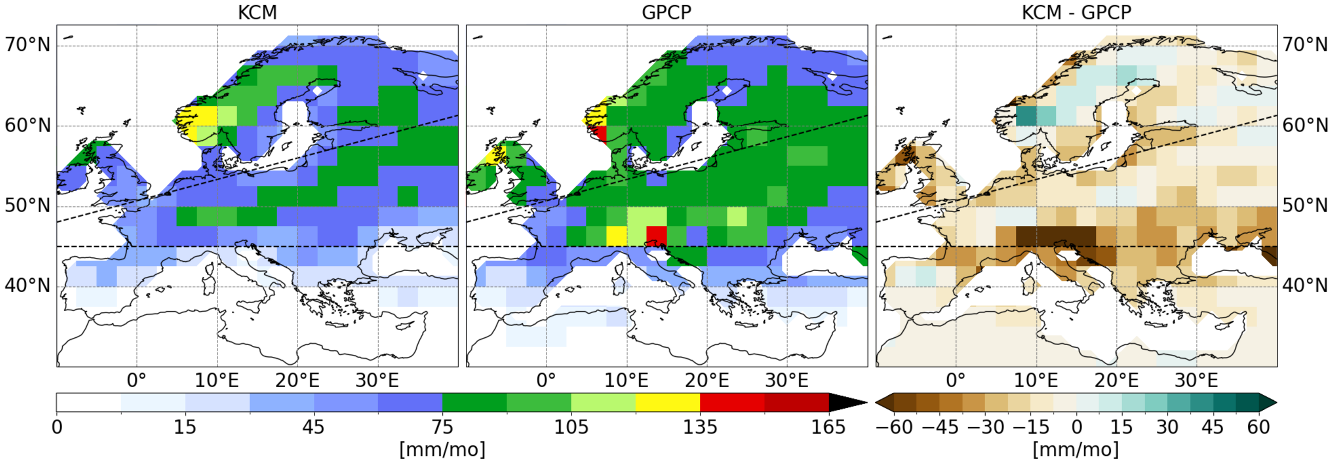

Supplementary Fig. 5 JJA average precipitation from (a) the reference period in the KCM (year 1-30), (b) the GPCP data set (year 1981-2010) and (c) bias (KCM – GPCP). Units are in mm/day

Soil moisture and water budget

Changes in soil moisture (in meters), h_{ws} are calculated using a single bucket method, with the maximum field capacity changing geographically

$$\rho_w \frac{\partial h_{ws}}{\partial t} = (1 - c_v)P_r + E_{ws} + M_{sn} + M_{snc} - R_s - \mathrm{D} \quad (1)$$

where ρ_w is the density of water, P_r is the rain, c_vP_r is the rain intercepted by the canopy, E_{ws} includes evaporation, evapotranspiration, and dew deposition, M_{sn} is the surface snow melt, M_{snc} is the excess snow melt in the canopy, R_s is the runoff, and D is the drainage (Roeckner et al. 2003). The three water fluxes studied in this paper are total precipitation, $P = P_r + P_{sn}$ where P_{sn} is snow precipitation, evaporation, $E = E_{ws}$, and runoff, which includes drainage, $R = R_s + D$.
